# Kaposi's Sarcoma-Associated Herpesvirus-Encoded LANA Down-Regulates IL-22R1 Expression through a Cis-Acting Element within the Promoter Region

**DOI:** 10.1371/journal.pone.0019106

**Published:** 2011-04-22

**Authors:** Ling Su, Qingjiao Liao, Yang Wu, Xulin Chen

**Affiliations:** State Key Laboratory of Virology, Wuhan Institute of Virology, Chinese Academy of Sciences, Wuhan, China; Karolinska Institutet, Sweden

## Abstract

Kaposi's sarcoma-associated herpesvirus (KSHV) is considered to be a necessary, but not sufficient, causal agent of Kaposi's sarcoma (KS). All forms of KS are characterized by the proliferation of spindle-shaped cells, and most (>90%) spindle cells from KS lesions are latently infected with KSHV. During KSHV latency, only a few viral genes are expressed. Among those latent genes, the ORF 73 gene encodes the latency-associated nuclear antigen (LANA), which is critical for the establishment and maintenance of the latent KSHV infection. Much evidence suggests that many cytokines can increase the frequency and aggressiveness of KS. In this study, a microarray analysis of KS and normal tissues revealed that multiple cytokines and cytokine receptors are regulated by KSHV latent infection. Of special interest, IL-22R1 transcript level was found to be down-regulated in the KS tissue. To study the possible regulation of IL-22R1 by LANA, the IL-22R1 promoter was constructed and found to contain a LANA-binding site (LBS). LANA was demonstrated to down-regulate IL-22R1 expression via direct binding to the LBS located within the IL-22R1 promoter region. Furthermore, KSHV latently infected cells showed an impaired response to IL-22 stimulation. These results suggest that LANA can regulate host factor expression by directly binding to a cis-acting element within the factor's promoter to benefit latent viral infection and suppression of the antiviral immune response.

## Introduction

Kaposi's sarcoma (KS) is a multicentric angioproliferative disorder that frequently involves the skin [1]. Kaposi's sarcoma-associated herpesvirus (KSHV) is considered to be a necessary, but not sufficient, causal agent of KS. KSHV is also associated with primary effusion lymphoma (PEL) and a subset of multicentric Castleman's disease [2]. KS can be subdivided into three clinical subtypes: cutaneous, mucocutaneous, and visceral types. All forms of KS are characterized by the proliferation of spindle-shaped cells, angiogenesis, inflammatory cell infiltration, and edema [3]. In early-stage KS, large numbers of inflammatory cells, including lymphocytes and macrophages, are recruited into KS lesions [4]. These cells produce high levels of proinflammatory cytokines and growth factors. Cytokines produced by inflammatory cells induce normal endothelial cells to acquire the features of KS spindle cells and to induce production of angiogenic factors [5]. Several cytokines and growth factors have been shown to support the growth of cultured KS spindle cells; these include IL-1β, IL-6, the soluble IL-6 receptor α, oncostatin M, and TNF-α [6], [7]. The evidence suggests that cytokines can increase the frequency and aggressiveness of KS by enhancing the effect of angiogenic factors or by reactivating KSHV reinfection, which is etiologically closely associated with KS [8].

Most (>90%) spindle cells from KS lesions are latently infected with KSHV, and only a few viral genes are expressed during KSHV latency [9]. Among those latent genes, the ORF 73 gene, which encodes the latency-associated nuclear antigen (LANA), is critical for the establishment of a latent KSHV infection. LANA is a large (1162 amino acid), multifunctional, constitutively expressed protein that is required for viral episome maintenance in proliferating cells [10]. Many researchers have found that LANA can function as a transcriptional modulator of various cellular and viral promoters, including its own promoter [11], [12], [13], [14], [15]. The activation of transcription by LANA is directed by many promoters containing binding sites for cellular proteins including ATF, AP-1, CAAT, or Sp1, which are linked to a TATA box [16]. LANA also contributes to broad repressive effects on transcription [17]. Although some of the transcriptional repression mediated by LANA occurs indirectly via interactions with corepressors including mSin3, SAP30, CIR, the methyl CpG-binding protein MeCP2, or the histone methyltransferase SUV39H1 [18], [19], [20], this viral protein inhibits TGF-β signaling through epigenetic silencing of the TGF-β typeαreceptor [21]. Direct binding of LANA to DNA has also been reported to result in the transcriptional repression of a viral gene [22].

IL-22R1 (interleukin 22 receptor 1α), whose alternative names include IL-22R, cytokine receptor family 2 member 9 (CRF2-9), is a 574 amino acid single-pass type I membrane protein belonging to the type II cytokine receptor family. IL-22R1-expressing tissues include barrier organs, lung, liver, kidney, colon and pancreas. IL-22R1 can form heterodimers with IL-10R2 or IL-20R2 and bind IL-22, IL-20, or IL-24 [23]. IL-22 is a member of the IL-10 cytokine family and is primarily produced by Th1, Th17, Th22 and NK cells [24], [25]. IL-22 first binds to the IL-22R1 extracellular domain with high affinity, and then IL-10R2 can sequentially recognize and bind to the IL-22/IL-22R1 binary complex [26]. This ternary complex activates the JAK/STAT signaling pathway, strongly activating STAT3 and weakly activating STATs 1 and 5, which leads to the diverse biological effects of IL-22. Furthermore, this complex could also activate the extracellular signal-regulated kinase (ERK), c-Jun N-terminal kinase (JNK), and p38 MAP kinase pathways in a rat hepatoma cell line [27]. IL-22 can be either pathogenic and inflammatory or protective depending upon affected tissue and the co-expression of inflammatory (IL-8 and CRP) or regulatory (SOCS 3, IL-10, and antibacterial peptides) molecules.

The present study demonstrates that several cytokines and cytokine receptors are regulated by KSHV latent infection, and that the transcription level of IL-22R1 was down-regulated in KS tissue compared with normal tissue. KSHV-positive cells also exhibited impaired responses to IL-22 stimulation. Furthermore, KSHV LANA was shown to be responsible for the down-regulation of IL-22R1 expression through direct binding to a cis-acting element that located within the IL-22R1 promoter region. Our results suggest that LANA may have evolved in multi-pathways to modify the cellular environment to benefit viral survival.

## Results

### Distinct expression levels of cytokines and cytokine receptors in KS lesions versus normal tissue

**Figure 1:**
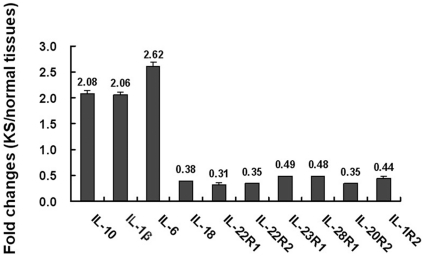
Expression levels of cytokines and cytokine receptors in KS lesions vs. normal tissues. A cDNA microarray analysis was performed to compare the gene expression between KS tissue and normal tissue. Differentially expressed interleukin-associated genes are listed (Ratio ≥2 or ≤0.5 and p value of log-ratio <0.05).

KSHV induces transcriptional reprogramming of infected cells [28] to adapt the environment for the benefit of the virus. To further identify cellular genes regulated upon the formation of a KS lesion, gene expression profiles were compared between Kaposi sarcoma and normal skin tissues by cDNA-microarray. KS tissue from a nodular lesion representing the most advanced stage of KS disease and normal tissue taken near the KS lesion were used. The analysis showed that a total of 2980 genes were significantly (p<0.05) up- or down-regulated (>2-fold) in the KS tissue compared to the normal tissue. In particular, a change in the expression levels of a subset of cytokines and cytokine receptors in the KS lesion was identified (Figure 1). IL-6, IL-1β, and IL-10 expression levels were all elevated more than two-fold; these cytokines have all been previously reported to be important contributors to the growth, survival, and spread of KSHV-associated disease [29], [30]. Notably, the gene expression level of IL-18, an IFN-γ inducing factor [31], was decreased 62%. We hypothesize that IL-18 may also be a key factor in KS pathogenesis.

Expression levels of several interleukin receptors, including IL-1R2, IL-22R1, IL-23R1, IL-28R1, IL-20R2, and IL-22R2, were dramatically down-regulated in KS tissue (Figure 1). Among these receptors, IL-22R1 and IL-20R2 can form a dimer to be recognized by IL-20 and IL-24 [23]. Recently, increasing data has shown that IL-20 and IL-24 play important roles in host inflammatory regulation [32], anti-angiogenesis [33], [34] and cell growth inhibition [35]. IL-22R1 was reported to drive inflammation in a mouse model [36]. Interestingly, the reduced expression of IL-22R1 in KSHV-infected cells was also reported by Wang et al. [28]. IL-22R2, a soluble, naturally occurring IL-22 antagonist, shares its amino acid sequence homology with IL-22R1 and is encoded by a gene physically adjacent to IL-20R1 [37]. IL-22R2 exhibited a similar reduced expression pattern, raising the possibility that the biological functions of IL-22 may be impaired in KS tissue. The down-regulation of IL-22R1 could be relevant to KSHV latency and pathogenesis.

### LANA down-regulates IL-22R1 promoter activity in a dose-dependent manner

**Figure 2:**
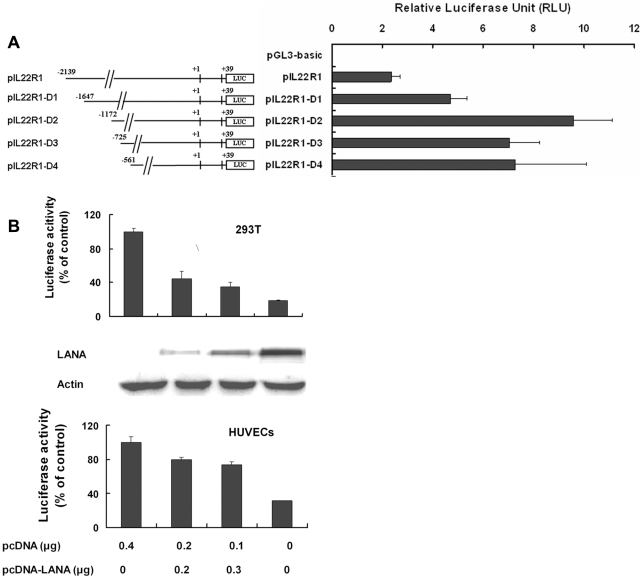
LANA down-regulates IL-22R1 promoter activity in a dose-dependent manner. (**A**) Schematic of pIL22R1 (−2139 to +39) and a series of deletion mutants (pIL22R1-D1, D2, D3, and D4). (**B**) LANA down-regulates IL-22R1 promoter (−2139 to +39) activity. pIL22R1 (−2139 to +39) was co-transfected with increasing amounts (0, 0.1, 0.2 or 0.4 µg) of LANA into 293T cells. At 48 h post-transfection, cells were lysed and assayed for luciferase activity. The expression levels of LANA were detected in cell lysates by western blotting using anti-LANA-C antibody (top panel). The regulation of the IL-22R1 promoter (−2139 to +39) by LANA was also analyzed in HUVEC cells (lower panel).

To investigate the regulation of IL-22R1 gene expression, a reporter plasmid (pIL22R1) was constructed by cloning the putative wide-type IL-22R1 promoter including the 5′ untranslated region (−2139 to +39, with +1 being the transcription initiation site) and inserting it upstream of the luciferase gene in the promoterless pGL3-basic vector. Transient transfection of pIL22R1 into 293T cells resulted in a greater than 40-fold increase in luciferase activity compared with the pGL3-basic vector control, demonstrating that this DNA fragment contains significant promoter activity (Figure 2A). Therefore, the IL-22R1 promoter pIL22R1 was fully functional. To further characterize the IL-22R1 promoter, a series of truncated mutants were constructed and all showed activity in 293T cells (Figure 2A). When an upstream promoter region (−2139 to −1172) was deleted, the promoter activity was found to elevate about 4-fold, indicating that a negative regulation element may exist in the region (Figure 2A). Similar results were also obtained in human umbilical vein endothelial cells (HUVECs) (data not shown).

Viral gene expression has been demonstrated to be highly restricted in KSHV latent infections, and most of the latency-expressed genes are important for maintaining viral latency by modulating various cellular pathways [2]. LANA has been demonstrated to regulate transcription [11], so we hypothesized that LANA could be responsible for the downregulation of IL-22R1. To test more possibilities in transcription regulation, we used a longer reporter, pIL22R1 (−2139 to +39), to do the following experiments. pIL22R1 (−2139 to +39) was co-transfected with different concentrations of full-length LANA expression vector pcDNA-LANA into 293T cells and HUVEC cells, and promoter activity was measured as relative luciferase units (RLU). As shown in Fig. 2B, the IL-22R1 promoter activity was repressed by LANA in a dose-dependent manner in both cells. The expression of LANA was confirmed in 293T cells and HUVECs by Western Blotting. Our results indicated that IL-22R1 can be transcriptionally down-regulated by the KSHV latent protein LANA.

### The LBS-like sequence is required for LANA to down-regulate IL-22R1 expression

**Figure 3:**
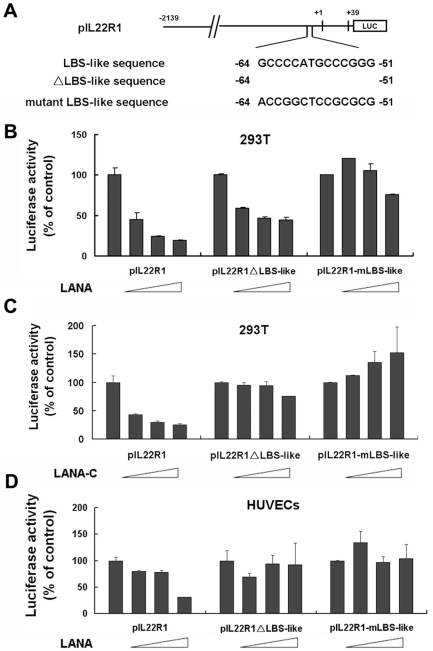
The LBS-like sequence is required for LANA to down-regulate IL-22R1 expression. (**A**) Schematic of pIL22R1 (−2139 to +39), pIL22R1ΔLBS-like and pIL22R1mLBS-like DNA sequences. (**B**) Increasing amounts of pcDNA-LANA expressing full-length LANA were co-transfected with either pIL22R1 (−2139 to +39) or its mutants (pIL22R1ΔLBS-like and pIL22R1mLBS-like) into 293T cells. At 48 h post-transfection, cells were harvested and assayed for luciferase activity. (**C**) Increasing amounts of pcDNA-LANA-C-expressing carboxyl-terminal domain (amino acids 951–1162) of LANA were co-transfected with either pIL22R1 (−2139 to +39) or its mutants (pIL22R1ΔLBS-like and pIL22R1mLBS-like) into 293T cells. At 48 h post-transfection, cells were harvested and assayed for luciferase activity. (**D**) Increasing amounts of pcDNA-LANA expressing full-length LANA were co-transfected with either pIL22R1 (−2139 to +39) or its mutants (pIL22R1ΔLBS-like and pIL-22R1mLBS-like) into HUVEC cells. At 48 h post-transfection, cells were harvested and assayed for luciferase activity.

LANA has been shown to specifically bind to two sites termed LANA binding site 1 (LBS1) and LANA binding site 2 (LBS2) within the terminal repeats of the viral genome. The core binding motif is GCCCCATGCCCGGGCG, and 13 of the 16 bp are conserved between the two sites [38]. To determine whether any LANA response elements were present in the IL-22R1 promoter region, a sequence analysis of the promoter region of the IL-22R1 gene was performed. An element (−64 to −48) with only one base pair mismatch (−50 T/C) to LBS1 was identified within the IL-22R1 promoter region. Due to the similarity in the nucleotide sequence, we hypothesized that LANA may have a potential binding activity to this element and this LBS-like sequence may play a role in the LANA-mediated repression of IL-22R1 expression. To confirm our hypothesis, we deleted nucleotides −64 to −51 from the pIL22R1 to construct pIL22R1ΔLBS-like (−64 to −51), and introduced substitution mutations in the LBS-like sequence to construct pIL22R1-mLBS-like (Figure 3A).

As shown in Figure 3B, LANA was able to repress the activity of the IL-22R1 WT promoter in a dose-dependent manner, whereas it had a much lower effect on the repression of pIL22R1ΔLBS-like and minimal effect on the activity of pIL22R1-mLBS-like. The binding domain of LANA to the LBS region has been reported to localize to residues 996–1139 [39]. When the pGL3-IL22R1 promoter was co-transfected with pcDNA-LANA-C (C-terminus of LANA, a.a. 951–1162), the wild type IL-22R1 promoter activity was shown to be down-regulated in dose-dependent manner (Figure 3C). However, LANA-C did not appear to down-regulate pIL22R1ΔLBS-like or pIL22R1-mLBS-like promoter activity (Figure 3C). These data strongly suggest that the down-regulation of the IL-22R1 promoter by LANA requires an LBS-like sequence and that LANA-C can bind to LBS and affect the regulation of IL22R1 expression similarly to full-length LANA. LBS-like sequence-dependent down-regulation of the IL-22R1 promoter was also observed to the same extent in HUVEC cells (Figure 3D). The expression of LANA and LANA-C were confirmed by western blot.

### LANA binds to the LBS-like sequence in the IL-22R1 promoter *in vitro*

**Figure 4:**
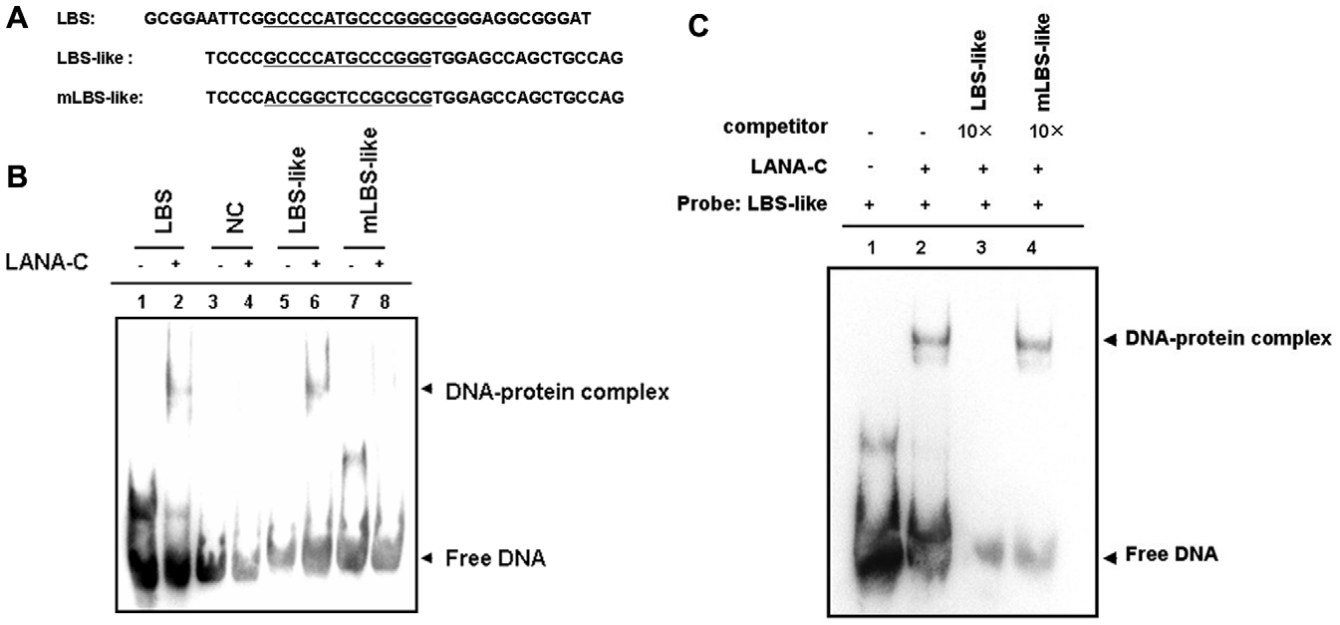
LANA binds to the IL-22R1 promoter in vitro. (**A**) The aligned sequences of DNA fragments from TR DNA, wild type and mutant IL-22R1 promoters which contain LBS, the wild type LBS-like sequence and scrambled-mutant DNA sequence (underlined) [38]. The nucleotide sequences represent the probe sequences used in the EMSA and were labeled with biotin. (**B**) LANA can bind to the LBS-like DNA sequence in IL-22R1 promoter. Probes as indicated were incubated for 20 min with or without purified LANA-C (a.a. 951–1162). NC, a DNA fragment derived from the sequence in M13 DNA, used as a negative control for LANA binding. (**C**) The binding of LANA to the wild type LBS-like sequence is specific. A 10-fold excess of cold competitor or mutant competitor DNA was added to compete the reaction between WT LBS-like and LANA. The upper and lower arrows indicate the LANA-specific binding band and free probe.

To test whether LANA can bind to the LBS-like sequence located in the IL-22R1 promoter, EMSA was performed. The His-tagged C-terminus of LANA was purified from *E.coli* BL21 cells that were transformed with pET-his-LANA-C. Three probes, LBS with a core LBS1 sequence (37 bp), an LBS-like region (−69 to −34) from pIL22R1 containing the LBS-like sequence, and a DNA fragment with a scramble mutation in the LBS-like sequence named mLBS-like, were designed and 5′-labeled with biotin (Figure 4A). Our results demonstrate that LANA-C bound to the IL-22R1 LBS-like sequence with an affinity similar to that of LBS1, which has been previously demonstrated to bind with LANA with high affinity [38] (Figure 4B, lanes 2 and 6). However, the LANA-C protein did not bind the negative control, a DNA fragment from M13 DNA or the probe bearing mutants in the LBS-like sequence (Figure 4B, lanes 4 and 8). Competition with the 10-fold excess unlabeled wild type probe (LBS-like fragment), but not with the mutated LBS-like sequence (mLBS-like), abolished the shifted band (protein-DNA complex) (Figure 4C, lanes 3 and 4), demonstrating that this complex represents a specific interaction between LANA and the LBS-like sequence.

### LANA binds to the LBS-like sequence in IL-22R1 promoter *in vivo*

**Figure 5:**
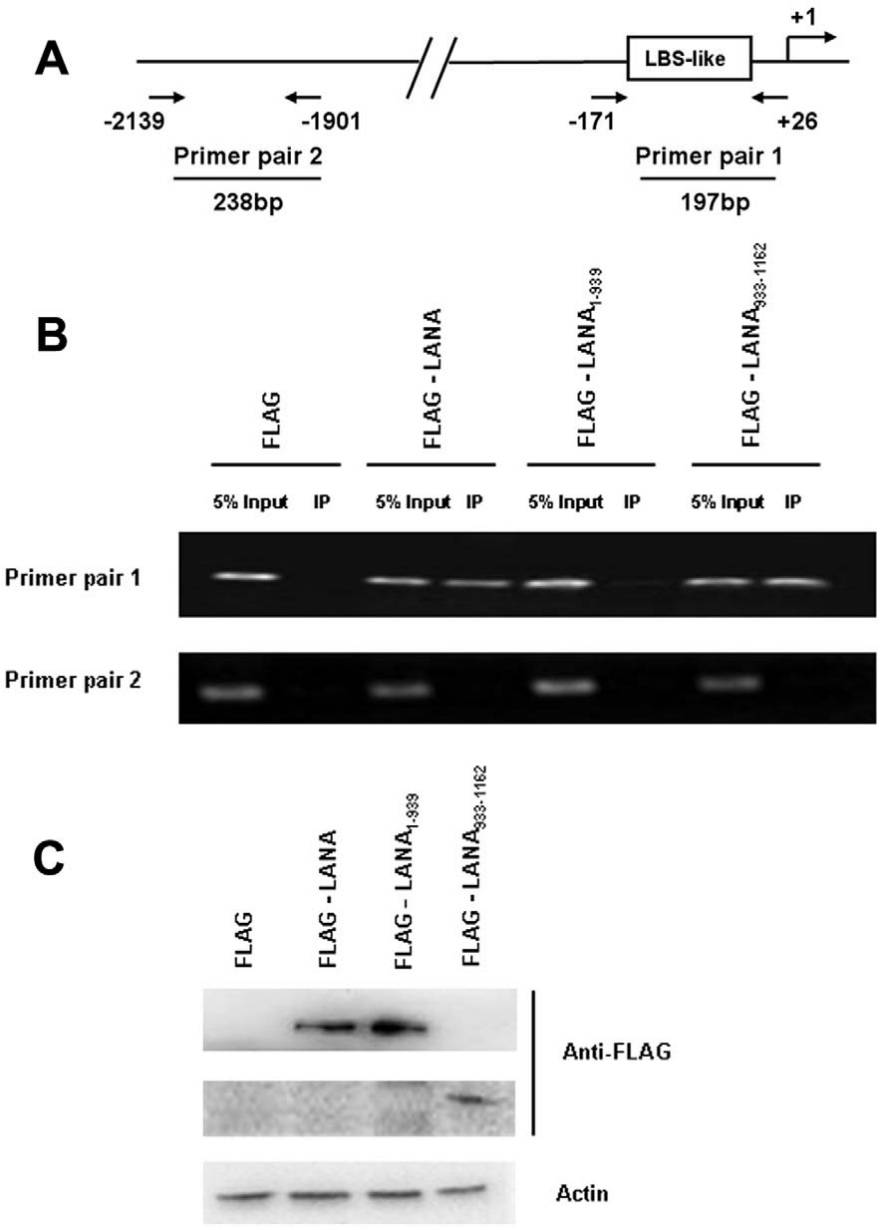
LANA interacts with the IL-22R1 promoter in vivo. (**A**) Schematic diagram showing the locations of the two pairs of primers used in the ChIP assay. (**B**) Formaldehyde cross-linked chromatin was prepared from 293T cells that were transfected with pFLAG, pFLAG-LANA, pFLAG-LANA_1–939_ or pFLAG-LANA_933–1162_, and immunoprecipitated with anti-FLAG antibody. PCR was performed with primer pair 1 to amplify a 197 bp DNA fragment containing LBS or with primer pair 2 to amplify a 283 bp DNA fragment, approximately 2 kb upstream of LBS-like sequence in IL-22R1 promoter. A sample representative of 5% the total input chromatin was included in the PCR analysis. (**C**) The expression levels of LANA and its truncated mutants were detected in cell lysates by western blotting using anti-FLAG antibody and anti-actin served as a loading control.

To further verify whether LANA can bind to the LBS-like sequence in the IL-22R1 promoter in vivo, ChIP assays were performed using an anti-FLAG antibody on samples from cross-linked 293T cells transfected with a full length LANA expression plasmid pFLAG-LANA or one of the two truncated forms of LANA, pFLAG-LANA_1–939_ and pFLAG-LANA_933–1162_. To amplify the ChIP signal, pIL22R1 was co-transfected in each experimental group. The cross-link reversed DNAs were PCR-amplified using the two primer pairs shown in Figure 5A. Primer pair 1 was designed to amplify a 197 bp sequence containing the LBS-like sequence. Primer pair 2 was designed to amplify a 238 bp sequence that was located approximately 2 kb away from the IL-22R1 promoter LBS-like sequence and was used as a negative control. As shown in Figure 5B, a band of 197 bp was PCR-amplified using primer pair 1 from the immunoprecipitated chromatin of the FLAG-LANA or the FLAG-LANA_933–1162_ expressing cell lysate, but not from the immunoprecipitated chromatin of the FLAG or FLAG-LANA_1–939_ expressing cell lysate. The 238-bp fragment could not be visualized in all groups. These data demonstrate that both the full-length and the C-terminus of LANA can bind specifically to the LBS-like sequence that is located within the IL-22R1 promoter region in vivo. Taken together, our results support the idea that the LANA protein is capable of binding to a cis-element located within the IL-22R1 promoter region.

### KSHV-infected cells show impaired response to IL-22 stimulation

Unlike other members of the IL-10 family, IL-22 can activate the JAK/STAT pathway, phosphorylating STATs 1, 3 and 5, and the three major MAPK pathways, inducing the phosphorylation of ERK1/2, JNK, and p38 kinase after interaction with the IL-22R1/IL-10R2 receptor complex [40]. To further investigate whether the function of IL-22 is impaired in KSHV-infected cells, the activation of STAT3 and ERK 1/2 in 293T and 293T-BAC36 cell line in response to IL-22 stimulation was assessed.

**Figure 6:**
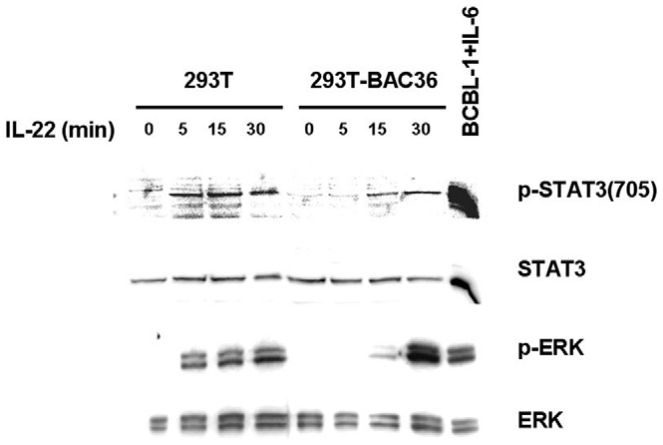
KSHV-infected cells show impaired response to IL-22 stimulation. 293T and 293T-BAC36 cells are stimulated with 100 ng/ml of rIL-22 for 0, 5, 15, or 30 min. The cells were lysed and subjected to immunoblot analysis to detect the phosphorylation of STAT3 and ERK.

Cells were collected at different time points after IL-22 was added to the culture media to a final concentration of 100 ng/ml. As shown in Figure 6, STAT3 and ERK 1/2 were activated in 293T cells only 5 min after treatment with IL-22. However, in 293T-BAC36 cells, in which BAC36 virions have established stable latent infection [41], it took 30 min of IL-22 treatment for a similar level of STAT3 and ERK 1/2 phosphorylation to occur. The BCBL-1 cell line treated with IL-6 was used as a control to monitor the activation of STAT3 and ERK 1/2 [42], [43]. These results suggested that KSHV-infected cells showed impaired responses to IL-22 stimulation, which may attribute to the decreased expression level of IL-22R1 in these cells.

## Discussion

KSHV is the causal agent of Kaposi's sarcoma (KS) and all forms of KS are characterized by the proliferation of spindle-shaped cells. The vast majority of spindle cells is latently infected and expresses only a small subgroup of viral proteins, including LANA [44]. In addition to tethering viral episomal DNA to the chromosomal DNA, KSHV LANA is well known as a transcription factor since it can activate as well as repress transcription. LANA activation of transcription is directed by multiple promoters containing binding sites for a range of cellular proteins that mostly linked to a TATA box [16]. And studies have shown that LANA can bind to various transcription factors like RING3 [45], ATF/CREB2 [17], CREB-binding protein (CBP), mSin3A [19] and glycogen synthase kinase 3 (GSK-3β) [46] to alter their function in modulating transcription. However, LANA is recently reported not a general processivity factor, as only those genes containing SRE elements can be activated by LANA [47]. Only a few LANA-responsive promoters have been identified and most of them are related to KSHV oncogenesis.

Using luciferase reporter assays, LANA was found to down-regulate the promoter activity of IL-22R1. When LANA was over-expressed in both 293T cells and HUVECs, IL-22R1 promoter activity was altered. Many evidences indicate that LANA is able to modulate transcription through two distinct mechanisms, interaction with upstream transcriptional regulators or direct binding of DNA. LANA has been shown to be able to down-regulate the expression of the virally-encoded K1 gene by directly binding to its promoter [22]. However, there was no data to demonstrate whether LANA can also regulate cellular gene expression by binding to the cellular chromosome DNA. The DNA sequence required for LANA binding has been identified as the LBS. By DNA sequence analysis, an LBS-like sequence which differs only in one nucleotide with LBS reported by Garber et al. [38], was identified in the 5′ −64 to −48 region upstream of the IL-22R1 gene. Our data further demonstrate that LANA can bind to this LBS-like sequence both in vitro and in vivo. Meanwhile, when the LBS-like region was mutated, the ability of LANA to down-regulate IL-22R1 was dramatically reduced. We have shown that the C-terminus of LANA (a.a. 951–1162) is responsible for LBS binding, and consistent with these observations, we observed that transiently transfected LANA-C alone can also reduce IL-22R1 promoter activity. The LBS-like sequence in the IL-22R1 promoter is located close to the transcription start site (−64 to −48), and the binding of a large protein like LANA may compete with other transcription factors to cause transcriptional repression. This is the first report indicating that LANA can bind directly to the host genomic DNA to regulate cellular gene expression. A previous report has also shown that LANA can silence TβR II gene expression by associating with the promoter of TβR II and leading to its methylation and to the deacetylation of the proximal histone. Indeed, KSHV LANA can modulate host gene expression in a multiple ways. The IL-22R1 promoter lacks a TATA box near its transcription initiation site but contains a Sp1-like element. Thus, Sp1 probably plays a role in the regulation of IL-22R1 expression. Previous reports have indicated that LANA can up-regulate survivin expression by forming a complex with Sp1 or Sp1-like proteins [48]. When the LBS-like region was deleted from the IL-22R1 promoter, we did observe the full length of LANA still can down-regulate IL-22R1 expression at a lower level, but LANA-C can not. So we speculate that Sp1 may also be involved in the regulation of IL-22R1 expression by LANA.

IL-22R1 belongs to the Class II cytokine receptors family (CRF2). The expression levels of IL-22R1 and IL-20R2, which can form a receptor complex to be recoginzed by IL-20 and IL-24, are found much lower in KS tissue than in normal tissue, suggesting that the function of the cytokines might be impaired in the KS lesion. IL- 20 is an anti-angiogenic cytokine [33], and IL-24 has also been confirmed as a potent inhibitor of angiogenesis. This effect is mediated by secreted IL-24 affecting endothelial cell growth through interactions with the IL-20/IL-22 receptor complexes [49]. IL-24 is also well known as potential anti-tumor drug [50]. IL-22 is another cytokine using IL-22R1 as its receptor. IL-22 is a member of the IL-10 family of cytokines produced by activated T cells and is involved in several tissue inflammation responses. The functional IL-22 receptor complex consists of two chains, IL-22R1 and IL-10R2. Although the IL-10R2 level did not show the same differences in our study comparing KS tumor tissue and normal tissue as IL-22R1, we did observe that KSHV-infected cells had impaired response to IL-22 stimulation. This result suggests that the reduced IL-22R1 levels may affect the function of associated cytokines. At least in early stage, KS lesions are thought to be angiohyperplastic-inflammatory lesions mediated by inflammatory cytokines and angiogenic factors[4]. We also hypothesize that low expression level of IL-22R1 exacerbates KS pathogenesis.

This report reveals that LANA down-regulates IL-22R1 expression through direct binding to a cis-acting element that located within the IL-22R1 promoter region. This is the first report to show that KSHV LANA can regulate host gene expression by directly binding to the cis-element within the promoter. This finding is important in understanding KSHV host interaction and viral pathogenesis.

## Materials and Methods

### Cell culture, antibodies and reagents

Human embryonic kidney cells 293T and 293T-BAC36 (293T cells harboring KSHV BAC36) were cultured in Dulbecco's modified Eagle's media (GIBCO/BRL) supplemented with 10% fetal calf serum at 37°C with 5% CO_2_, and 200 µg/ml of hygromycin B was added to the 293T-BAC36 culture media. HUVECs, human umbilical vein endothelial cells, were grown in M199/EBSS culture media (Thermo) supplemented with 10% fetal calf serum at 37°C with 5% CO_2_. Cells were stimulated with human recombinant IL-22 (Cell Signaling Technologies) for the indicated times. Rabbit anti-LANA antibody recognizing the C-terminus of LANA (amino acid 951–1162) was prepared in our laboratory. Anti-total STAT3, anti-phosphorylated STAT3 (Ser705), anti-ERK and anti-phosphorylated ERK antibodies were purchased from Cell Signaling Technologies. Anti-IL-22R1 antibody was purchased from Santa Cruz. Anti-Flag antibody (M2) and anti-actin antibody (AC-15) were purchased from Sigma.

### Plasmids

Eukaryotic expression plasmids pcDNA3.1-LANA and pcDNA3.1-LANA-C (a.a. 951–1162) were generated to express full-length and the C-terminus of LANA, respectively. The DNA fragment containing the LANA open reading frame was amplified with the primers 5′-CCCCCCAAGCTTATGGCGCCCCCGGGAATG-3′ (forward), 5′-CCGCCGGAATTCTTATGTCATTTCCTGTGGAGAGTC-3′ (reverse) for full-length LANA and 5′- GCGCCCAAGCTTATGGATTACCCTGTTGTTAGCACA-3′ (forward), 5′-GGCCGCGAATTCTTATGTCATTTCCTGTGGAGA-3′ (reverse) for LANA-C by PCR from BCBL-1 Hirt-DNA and were then cloned into the *Hind*III and *EcoR*I sites of the pcDNA3.1 plasmid. pSG5-FLAG-LANA was a gift of Kenneth M. Kaye (Harvard University) [10]. FLAG tagged truncated LANA mutant cDNA were amplified using PCR and cloned into pCMV-FLAG to produce FLAG-LANA_1–939_ (1–939 a.a.), FLAG-LANA_933–1162_ (939–1162 a.a.). Primer 5′-CCAAGCTTATGGCGCCCCCGGGAATGCGCCTG-3′ (forward), 5′-CGGAATTC TTA TGTCATTTCCTGTGGAGAGT-3′ (reverse) for amplifying LANA_1–939_ and 5′-CCAAGCTTATGGCGCCCCCGGGAATGCGCCTG-3′ (forward), 5′-CGGAATTCTTACAAGATTATGGGCTCTTCCACCGT-3′ (reverse) for amplifying LANA_933–1162_. The reporter plasmid pIL22R1 was constructed by cloning the 5′ untranslated DNA sequence of IL-22R1 −2139 to +39 (+1 being the initiation site) into the pGL3-basic vector (Promega) at the 5′ *Mlu*I site and the 3′ *Xho*I site. This fragment was PCR amplified using BJAB genomic DNA as the template. pIL22R1ΔLBS-like and pIL22R1-mLBS-like were constructed by inserting an *EcoR*I/*Xho*I ΔLBS (lacking the 14 bp LBS-like sequence ) or mutant LBS fragment containing a random rearrangement of the 14 bp LBS-like sequence (ACCGGCTCCGCGCG) to replace the *EcoR*I/*Xho*I fragment of pIL22R1. A series of truncated mutants of pIL22R1 were also constructed by PCR, and the fragments were inserted into pGL3-basic. Primers used in construction of luciferase plasmids are shown in Table 1. These constructs were all confirmed by DNA sequencing (Invitrogen).

**Table 1:** Sequences of the primers used for the generation of luciferase reporter plasmids.

Primer Name	Sequence (5**′**-3**′**)
pIL22R1(+)	CGACGCGTCAAGCAATTCTCCTGCTTCAGC
pIL22R1(−)	CCGCTCGAGCGGGGCTGGCACAGAGCCCTCCC
pIL22R1-D1(+)	CGACGCGTGCTTCCCCACACTGCCTTGTCATCA
pIL22R1-D2(+)	CGACGCGTAAATGACTGGCATGGGCCCTACCTT
pIL22R1-D3(+)	CGACGCGTATGAAGTTCAGCCCCATCTCTAGCA
pIL22R1-D4(+)	CGACGCGTACCCCAAGCGTGGGGACCTGCCTTG
mLBS-like(+)a	GCGGAATTCCAGAGCACACAGGGCCAGGAC
mLBS-like(−)	CACGCGCGGAGCCGGTGGGGAGAAGGGGGTGGGTGGGAC
mLBS-like(+)b	CCACCGGCTCCGCGCGTGGAGCCAGCTGCCAGGGCGCCA
ΔLBS-like(−)	GCTGGCTCCAGGGGAGAAGGGGGTGGGT
ΔLBS-like(+)	CCTTCTCCCCTGGAGCCAGCTGCCAGGGCG

Restriction enzyme sites are underlined.

### Transient transfection and western blot analysis

All plasmids for transfection were purified by QIAGEN miniprep kit. Transfection of HUVEC cells and 293T cells was performed using the Lipofectamine 2000 reagent (Invitrogen) according to the manufacturer's instructions.

Protein expression levels were measured by western blotting. At the time of harvest, cells were washed by ice-cold PBS (phosphate-buffered saline) and lysed in RIPA lysis buffer. Lysates were prepared and subjected to sodium dodecyl sulfate-polyacrylamide gel electrophoresis (SDS-PAGE), followed by electrotransfering onto immobilon-p polyvinylidene difluoride membranes (Millipore, Bedford, MA). The membranes were incubated in blocking buffer (50 mM Tris-HCl [pH 7.4], 0.2 M NaCl containing 5% non-fat milk and 0.1% Tween) for 1 h followed by incubation with primary antibodies overnight at 4°C. The membranes were then washed with TBS-T and incubated with HRP-conjugated goat anti-mouse or anti-rabbit antibody (Thermo) for 1 h at room temperature. Signals were detected with enhanced-chemiluminescence substrate (ECL; Thermo) and an AlphaEase® FC Imaging System (Alpha Innotech Corporation).

### EMSA

Electrophoretic mobility shift assay was carried out as described by Shin & Park [51] with modifications. The double-stranded oligonucleotides were prepared by annealing sense and anti-sense oligonucleotides which were 5′ end-labeled with biotin. Each binding reaction mixture containing 20 mM Tris/HCl (pH 7.4), 100 mM NaCl, 4 mM MgCl_2_, 0.25 mM EDTA, 1 mM dithiothreitol, 0.5 µg poly (dI-dC), 10% (v/v) glycerol, 100 ng labeled oligonucleotide and 0–400 ng purified LANA-C (a.a. 951–1162) protein. After incubation at room temperature for 20 min, reaction mixtures were separated on 6.5% native polyacrylamide gels. Electrophoresis was performed in 0.5× TBA (Tris-borate-EDTA) at 100 V on ice. The DNA-protein complexes were transferred onto Hybond-N^+^ membranes (Amersham) and were immunoblotted with HRP-conjugated streptavidin. Signals were examined with enhanced-chemiluminescence substrate (ECL; Thermo) and an AlphaEase® FC Imaging System (Alpha Innotech Corporation).

### Chromatin immunoprecipitation (ChIP) assay

A total of 5×10^6^ 293T cells were transfected with 12 µg of pCMV-FLAG, pSG-FLAG-LANA, pFLAG-LANA1_-939_, or pFLAG-LANA_933–1162_. At 48 h post-transfection (p.t.), the cells were collected and treated with formaldehyde for 10 min followed by the addition of glycine to a final concentration of 0.125 M. Cells were then washed twice with cold PBS and were resuspended in lysis buffer (1% SDS, 10 mM EDTA, 50 mM Tris-HCI, pH 8.1) with protease inhibitors. After brief sonication to fragment the DNA to an average fragment size of 200–1000 bp, the DNA fragments cross-linked to proteins were enriched by immunoprecipitation with an anti-FLAG antibody overnight at 4°C. After reversal of the cross-linking and DNA purification, the extent of enrichment was monitored by PCR amplification. PCR was run at 95°C for 30 s, 55°C for 35 s, and 72°C for 1 min. The primers for the LBS region were 5′-CTCCCTTAGATCCCGCCCAGAACCT-3′ (forward) and 5′- GAGCCCTCCCTTGGCCTCTACTC-3′ (reverse). The primers for the non-LBS fragments used as negative control were 5′-GCAATTCTCCTGCTTCAGCCTCCCG-3′ (forward) and 5′- GAACACCCAGACTTCATTTCTAACA-3′ (reverse).

### Luciferase assay

Luciferase reporter plasmids, pRL-TK plasmid, pcDNA3.1-LANA, pcDNA3.1-LANA-C, or pcDNA3.1 empty vector were mixed and transfected into cells. After 48 h, the cells were collected. A luciferase assay was performed with the Dual Luciferase Reporter Assay System (Promega) according to the manufacturer's protocols. The relative values of firefly luciferase activity were determined by normalizing to Renilla luciferase activity for transfection efficiency.

### cDNA microarray and data analysis

Total RNA of frozen sections of AIDS-KS lesions and surrounding normal tissue from the same patients were extracted using TRIzol reagent (Invitrogen), followed by purification on an RNeasy column (Qiagen) and quantified by UV absorption (Nanodrop). RNA quality was assessed with a 2100 bioanalyzer and the RNA 6000 LabChipR (Agilent Technologies). The SBC homemade human cDNA microarray (Shanghai, China) containing 15552 spots (including 768 controls and 14784 probes) was used to evaluate the expression level of 10379 known genes and 3022 ESTs. The microarray was made as previously described (Huang et al., 2006). An Agilent Low RNA Input Fluorescent Linear Amplification Kit (Agilent Technologies) was used for RNA linear amplification following the manufacturer's protocol. RNA samples from normal tissue were pooled and used as a reference labeled with Cy3 (GE Healthcare). RNA from KS tissue was isolated in triplicate and labeled with Cy5 individually. Cy3- and Cy5-labeled cRNA pools were mixed to hybridize to the microarrays. Hybridization and washes were performed using a standard protocol followed by scanning on an Axon 4000B Scanner (Axon Instruments). Microarray raw data intensity profiles were analyzed using GeneSpring 7 (Agilent technologies) to perform locally weighted scatter plot smoothing (LOWESS) normalization and statistical analysis. The normalized data were then subjected to zeroing and normal tissue samples served as the zeroes. Changes in gene expression were considered significant if the detection *P* value was less than 0.05 (log-ratio p value<0.05) and the magnitude of the change was at least 2-fold.
